# Implementation of the European School Fruit and Vegetables Scheme in Spain (2009–2017)

**DOI:** 10.3390/ijerph16203898

**Published:** 2019-10-15

**Authors:** Panmela Soares, Iris Comino, María Asunción Martínez-Milán, M. Carmen Davó-Blanes, Cesare Altavilla, Pablo Caballero

**Affiliations:** 1 Department of Community Nursing, Preventive Medicine and Public Health and History of Science, University of Alicante, 99-03080 Alicante, Spain; panmela.soares@ua.es (P.S.); mariasuncion.m.m@gmai.com (M.A.M.-M.); mdavo@ua.es (M.C.D.-B.); eatingfaster@gmail.com (C.A.); pablo.caballero@ua.es (P.C.); 2 Public Health Research Group, University of Alicante, 99-03080 Alicante, Spain

**Keywords:** child, fruit, vegetables, school health services, public policy, environment and public health

## Abstract

The School Fruit and Vegetables Scheme (SFVS) implemented by the European Union in 2009/2010 aims to improve the diet of students and to support agricultural markets and environmental sustainability. The objective of this study was to identify the characteristics of the School Fruit and Vegetables Scheme implementation from 2009 to 2017 in Spain and its autonomous communities. A descriptive, longitudinal, observational, and retrospective study was carried out on the basis of document analysis of SFVS reports. We studied the average budget for Spain and its autonomous communities (AC), the number of students enrolled, the cost of the SFVS by student and by day, the duration of the SFVS, the quantity of fruits and vegetables (FV) per student and day (g), the variety of FV, the recommendation to include local, seasonal, and organic foods, and the educational activities (EA). The results were studied by the AC which are territorial entities of Spain. The budget almost doubled during the study, thanks mainly to EU funds. However, the number of students increased only from 18% in 2009 to 20% in 2016. The quantity of FV increased from 2579 to 4000 tons, and the duration of the SFVS increased from 9.8 to 19.6 days. In the AC, there were variations in EA, in the number of enrolled students (7.4% to 45.6%), in the cost per student (from €2.3 to €28), and in the duration in days (5.6 to 70 days). The recommendation to include local, seasonal, and organic foods was implemented in five of the eight years studied. The development and scope of the SFVS in Spain are still insufficient to generate an equitable healthy dietary pattern in the school population. However, the SFVS has generated an economic market for agricultural production due to the amount of FV distributed in each academic course.

## 1. Introduction

The low intake of fruits and vegetables (FV) is an important risk factor for the development of non-transmissible diseases [1,2]. Promoting their consumption from infancy is a priority for public health thanks to their ability to lower the burden of disease [3].

From this perspective, the European Commission (EC) recognizes the importance of developing a plan to promote the consumption of fruit and vegetables in schools, supported by the EU [4] (Commission of the European Communities, 2007), and in the 2009–2010 academic course, the School Fruit and Vegetables Scheme (SFVS) was implemented. The SFVS aims to act on health, diet, agricultural markets, social equality, and regional cohesion [5,6].

Member states participating in the SFVS may choose the appropriate geographical and administrative level at which they wish to apply the SFVS. The appropriate administrative level at which to apply the SFVS in Spain is the autonomous community level, mainly for reasons related to the delegation of responsibilities within the Spanish State. The competencies in agricultural, health, and education matters are transferred to the autonomous communities (AC) which are administrative-territorial entities. For these reasons, the SFVS is carried out at the regional level, although the competent national authority establishes a common framework. Therefore, AC establish, in their respective regional strategies, how and when to distribute the produce. The EC considers the distribution of produce at mealtimes (school canteen) not to be appropriate for three fundamental reasons: control difficulties, inaccessibility of some students to the school canteen, and the possibility of replacing the item with other menu options [5,6]. 

The SFVS receives funding from the European Commission, central government, regional governments (autonomous communities) and, in some cases, private companies [7]. The budget allocated to the SFVS is granted for the distribution of FV to the group target in the affiliated schools, for a series of educational activities (EA) designed to stimulate healthy eating habits in the child population in the short and long term [5,7,8] and for logistics and distribution costs, equipment, communication, monitoring, and evaluation of the SFVS.

For SFVS implementation, authorization requests are solicited annually in accordance with the conditions established by the competent authority of the autonomous community. The State can select schools that meet specific conditions [5].

Following the international recommendations for promoting a healthy diet [2,9], the SFVS is an integral and coordinated strategy among different sectors, which promotes agricultural production of FV while stimulating consumption in schools [6]. This set up gives it the potential to influence different determinants of health such as education, environment, agriculture, and employment [10]. The SFVS aims to improve the eating habits and also to support the distribution of local and seasonal products using short commercial chains with active participation of agricultural producers [4]. Using this distribution formula for foods has been identified as a strategy that contributes to promoting a more sustainable and healthy food system [11,12,13].

Currently, in Spain, the SFVS is being carried out in different AC in a coordinated way between the Ministry of Agriculture, Fishing, and Food (MAPA), the Ministry of Health, Social Services, and Equality, and the Ministry of Education, Culture, and Sports [8]. However, although the SFVS has been implemented since 2009, there is little information on how it has been carried out in the different autonomous communities and on whether there are differences between them related to social equality and regional cohesion. Given the potential of the SFVS to support more sustainable and healthy food systems, its implementation and development in Spain continue to be important. The objective of this study is to identify the characteristics of the School Fruit and Vegetables Scheme implementation from 2009 to 2017 in Spain and its autonomous communities.

## 2. Materials and Methods

We carried out a descriptive, longitudinal, observational, and retrospective study nationally based on secondary sources. Sources included all the annual SFVS plans in Spain from 2009/2010 to 2016/2017, available on the webpage of MAPA [7,14,15,16,17,18,19,20].

The following variables were extracted from the reports consulted and were calculated by academic course (school time period between September and June) and AC: European and state budget (€), number of students enrolled (*n*), duration of the SFVS (days), quantity of FV included (*t*), varieties of FV (*n*), recommendation of local foods (yes, no), seasonal foods (yes, no) and organic foods (yes, no), and education activities.

For each AC and academic course, we calculated the average budget financed by the EU and by state (€, %), the number of students enrolled in the SFVS (*n*,%), the cost of the SFVS per student and per day (€), the duration of the SFVS (days), the quantity per student (kg), the quantity per student and day (g), and the variety of FV (*n*). Also, we studied the correlation between cost per student and recommendation of local, seasonal, and organic foods. In order to calculate the average number of students enrolled, the average cost per student, and the average quantity of FV per student, the values were weighted by the number of students enrolled in the SFVS in each studied autonomous community. To calculate the percentage of students covered by the SFVS, we used the target group of students enrolled in each AC between 3 and 18 years of age, as indicated in the regulations [5,21,22]. To calculate the cost per student, we divided the budget of each autonomous community among the students that adhered to the SFVS in that autonomous community. The grams per student in terms of the duration of the SFVS is the amount of FV offered per day and student. The data are presented for the whole of Spain by academic course and by AC. In the case of AC, the number of times that local, seasonal, and organic foods were included is also shown.

Also, with the objective of exploring the varieties of FV that were most frequent in the SFVS, we accounted for the number of times that each fruit or vegetable was included in the SFVS in each AC.

To know the educational strategies used by each AC in the SFVS, the EA were grouped into six categories: (1) Playful educational activities (campaigns, contests, workshops, games, theatrical performances and exhibitions on food habits, merchandising, FV calendars, gymkhanas, comics and animations about FV consumption, miniseries); (2) Didactic material (posters, brochures, cards, posters, teaching units, pedagogical guides, web resources); (3) Visits to the field and/or to farms, fruit and vegetable producers, and marketers of FV; (4) Training sessions (lectures, talks, colloquia for teachers, students, and parents); (5) Cooking workshops (cooking competitions, preparation of dishes with FV, tasting of products, sensory tastings, and preparation of recipes with fruit); (6) School gardens that included agricultural workshops and gardening sessions. The number of times each of them was present in the SFVS was counted by the autonomous community and academic course, and the first year of incorporation was identified.

## 3. Results

Table 1 describes the SFVS in Spain from 2009 to 2017. During the eight years studied, the SFVS was implemented in 14/15 autonomous communities of the 17 that make up Spain. During the first year, SFVS coverage reached 18.6% of the students. Although this figure fell in 2013 (15.6%), it increased again in the following years, reaching 20.5% in the 2015/16 academic year. The cost per student went from €7.8 in 2009/2010 to almost €10 in the last year and had a notable reduction in the 2012–2014 period. The duration of the SFVS, that is, the days when the SFVS was implemented grew gradually, with an average of 9.8 days in 2009/2010 rising to 19.6 days in the 2016/2017 academic year. The average cost per student and day was reduced across the entire period, from €1.80 in 2009/2010 to €0.51 in 2016/2017. The offering of FV also increased, reaching 4000 tons (2.8 kilos per student) in 2016/2017. The variety of fruits distributed remained constant, approximately 21 kinds of fruits, whereas the variety of vegetables increased from 5 to 10 (between 2009/2010 and 2016/2017).

**Figure 1:**
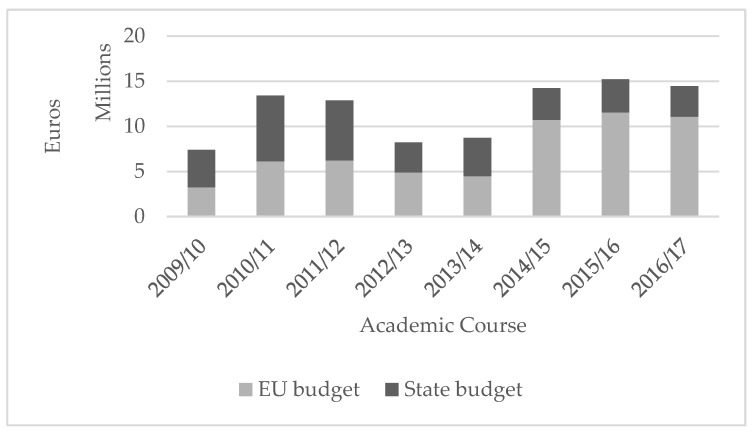
Annual distribution of budget supplied by the EU and the country of Spain for the implementation of the School Fruit and Vegetables Scheme.

Figure 1 shows the annual distribution of the country and the EU budget for the SFVS in Spain. In 2009, financing came mainly from country funding. In the two following years, there was an increase in the country and European budgets. In the 2012–2014 period, both budget sources decreased, with the reduction from the country fund being more pronounced. In the last period of 2014–2017, the allocation of country funding continued to fall; however, the global budget increased due to the funds allocated by the EU.

Table 2 shows the characteristics of the SFVS in the autonomous communities during the 2009–2017 period. Except for Madrid, which did not join the SFVS, the other autonomous communities participated in the program for a number of editions between three (Cantabria and País Vasco) and eight during the period studied. Andalucía and Cataluña had a higher average budget than the rest of the autonomous communities. However, the SFVS coverage for students was higher in Cantabria (64%) and Castilla y León (45.6%). The average cost per student was higher in Navarra (€28.4) and La Rioja (€19.3). Both communities also had a longer average duration of the SFVS (69.9 and 70.8 days, respectively). Galicia, Cantabria, and Navarra were the autonomous communities with the lowest average cost of SFVS per student per day, (€0.20, €0.30, and €0.50, respectively). Fruit and vegetable rations provided per day and student varied from 393 g (Andalucía) to 70 g Murcia. The autonomous communities that declared the highest amounts of fruits and vegetables per student per year were Navarra (8.1 kg) and Islas Canarias (5.1 kg). Regarding the variety of fruits, there was great variability among the autonomous communities: from 13.4 varieties in Cataluña to 1.9 in Navarra. Vegetables were not included in four of the AC (Aragón, Asturias, Galicia, and País Vasco). Except for Aragon and Navarra, the rest of the autonomous communities incorporated local foods in at least two editions of the SFVS from the 2012/2013 academic year. Seasonal foods were incorporated in all of the AC in some edition. However, 7 of the 16 AC did not include organic foods in any edition of the SFVS. There were no significant differences between the cost per student and the recommendation of local, seasonal, and organic foods.

**Figure 2:**
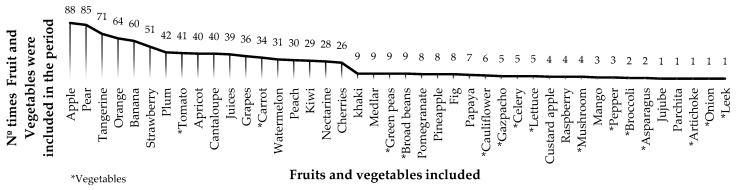
Frequency and kinds of fruits and vegetables included in the School Fruit and Vegetables Scheme in Spain (2009–2017).

Figure 2 shows the frequency and kinds of fruits and vegetables included in the SFVS in the whole of Spain. The frequency and variety of fruits were more predominant than those of vegetables. The most frequent fruits were apples and pears (88 and 85 times, respectively), and the most frequent vegetables were tomatoes and carrots (41 and 34, respectively).

Finally, Table 3 shows the EA included in the strategies of the different autonomous communities. Andalusia and Murcia stood out for their greater variety of EA in the different editions of the SFVS, and Cantabria for not having any. Recreational-educational activities, elaboration of didactic materials, and visits were the most frequent activities in AC during the 2009–2017 period. Andalusia was a pioneer in the implementation of educational activities to accompany the SFVS compared to the rest of the autonomous communities. By 2009, this community had declared recreational-educational activities, the elaboration of didactic materials, and training days and in later editions, it incorporated cooking workshops and school gardens. Visits to agricultural centers or vegetable gardens as well as fruit and vegetable producers and marketers were reported for the first time in Catalonia and Murcia in 2010/2011.

## 4. Discussion

The purpose of this study was to identify the characteristics of the School Fruit and Vegetables Scheme implementation from 2009 to 2017 in Spain and its autonomous communities. The majority of the AC participated in the different editions of the SFVS organizing the distribution of FV along with various educational activities. Although the SFVS had increasing coverage in terms of the students, its scope was limited. In addition, the implementation of the SFVS in each of the AC was very heterogeneous. The SFVS received more and more financial support from the European Union for its implementation, while funding at the state level was progressively reduced. Even so, the quantities of fruits and vegetables provided in the SFVS increased. This, together with the incorporation of local, seasonal, and organic foods in some autonomous regions is in accordance with the strategies to promote a healthy diet and a more sustainable food system.

The participation of a large proportion of the autonomous communities in the different editions of the SFVS shows their interest in incorporating the international recommendations in their educational offerings to promote the consumption of fruits and vegetables among the student population. Specifically, childhood is the ideal time to establish healthy eating behaviors, since these will probably persist in adult life [23,24,25].

The availability of fruits and vegetables in the school environment seems to encourage consumption among children [26,27,28], which helps to reduce the intake of unhealthy foods [29,30]. However, the amounts of FV provided to the students in the SFVS are too low. Only four ACs offered rations bigger than 144 g as recommended by the Scientific Committee of “5 per day” [31]. This is important, since there are students that only have the opportunity to eat FV in school time.

Furthermore, the distribution of fruits and vegetables alone is not sufficient to establish a healthy eating pattern [28,32,33,34]. Other complementary actions are required to increase information and raise awareness of the benefits of their consumption [33,34,35,36]. Thus, SFVS should be accompanied by educational activities. In line with this demand, our results showed a large number and variety of educational activities.

However, a large number of theory-based training activities (didactic material and training sessions) that are being developed may limit the scope of the educational objectives of the SFVS, given that they emphasize the conceptual objectives of the SFVS more than changing attitudes or procedures. In fact, previous studies show that with recreational–educational activities and cooking workshops, better results are achieved [35,36,37,38]. In addition, the potential benefits of SFVS to improve the dietary pattern of students can be compromised by the high variation observed in the different autonomous regions and the program sustainability [39,40]. As our results show, although SFVS coverage increased throughout the period studied, its scope was limited both by the number of days devoted to its implementation and by the number of students enrolled. These results suggest a possible limitation in the scope of the SFVS’ objectives, that is, to promote changes in the dietary pattern.

Furthermore, the heterogeneous development of the SFVS in the autonomous communities observed in the variables studied suggests unequal access to the program for the students according to their place of residence. This could lead to greater inequality in health, especially in families with low incomes and difficulties in accessing adequate food supplies [26,29,30]. This heterogeneity could be explained by its management by the autonomous communities and the absence of a sufficiently structured common regulatory framework. The predominance of the European Union funding of the SFVS over that received by the state suggests the existence of a disconnect between the actions carried out by both actors. The development and implementation of policies to promote healthy eating in schools require integrated efforts from different sectors [9,11]. Despite the interest shown by the Spanish state in promoting a healthier diet through other initiatives such as the NAOS strategy (Nutrition, Physical Activity, and Prevention of Obesity) [41], the Perseo Program (Guide for an Active Healthy School) [42], and the THAO program (prevention of childhood obesity based on actions in municipalities) [43], these initiatives have been carried out in parallel with the SFVS but in a disjointed way.

The progressive increase in the quantities of fruits and vegetables provided in schools can favor the development of agricultural markets. That is why institutional buying creates a new market for family farmers. Prioritizing the purchase of local, seasonal, and organic foods would contribute to a more sustainable food system [44,45]. In addition, this would be in line with the recommendations of the European Commission to reverse the negative impact of the current food production system on the environment and society [46]. Our results show that including local, seasonal, and organic food into the SFVS does not increase the cost per student. Even so, the incorporation of these recommendations in the SFVS is still just beginning.

When interpreting these results, we must remember that the information used came from the annual reports of the SFVS, prepared by the strategy managers in each AC. This can result in certain limitations due to the use of secondary data; as such, we do not have de control of the collection of these data. Also, we have no information on which schools implemented better the SFVS in each AC. However, the reports present the information in a homogeneous way for the different academic courses studied, which allowed us to explore how the SFVS is implemented in Spain. On the other hand, although this study focuses on a single country (Spain), which makes it difficult to extrapolate and generalize the results, the proposed methodology allowed us the describe some characteristics of the implementation and evolution of the SFVS. However, we do not have information about whether the availability of FV was equal in all schools which adhered to the SFVS. This is the first study to explore the scope of the SFVS. Given that the SFVS is an EU strategy, this study can contribute to decision-making to strengthen or introduce changes in the SFVS.

## 5. Conclusions

The development and scope of the SFVS in Spain are still insufficient to generate an equitable healthy dietary pattern for the students. The SFVS covers a limited percentage of the target group of students and is carried out heterogeneously in the different autonomous communities. This finding is contradictory to the purpose of the SFVS, that is, to improve the dietary pattern of students regardless of their geographic and socioeconomic status. Also, the continuity of the program depends largely on the funds that come from the EU. However, the SFVS has generated a consumer market for agricultural production. This information is related to the fact that, according to the data presented, thanks to the SFVS, a great amount of FV is being distributed. Before the implementation of the SFVS, these quantities were not acquired by schools. Taking into account the potential of the SFVS to improve children’s food consumption in line with sustainable development objectives, it is important to guarantee its implementation. Increasing the Spanish portion of the budget and promoting synergies among the agents involved could improve the coverage and duration of the SFVS, which would help promote sustainable food systems.

## Figures and Tables

**Table 1:** Characteristics of the School Fruit and Vegetables Scheme in Spain: averages for the 2009–2017 period.

Academic Course	AC	Students (millions)	Cost per Student/Year	Duration	Cost per Student/Day	Quantity of FV Included	Quantity of FV per Student	Variety	
								F	V
	*n*	*n* (%)	€	days	€	*t*	kg	*n*	*n*
09/10	14	1.29 (18.6)	7.8	9.8	1.80			20	5
10/11	14	1.28 (18.2)	8.2	12.4	0.66			20	7
11/12	15	1.32 (18.5)	8.3	10.2	0.81	2579.5	1.9	22	11
12/13	15	1.28 (17.9)	6.4	11.2	0.57	3992.8	3.3	21	7
13/14	14	1.12 (15.6)	6.9	12.3	0.56	2663.4	2.4	21	8
14/15	14	1.44 (19.9)	7.3	11.0	0.66	3068.8	2.1	17	9
15/16	14	1.49 (20.5)	7.8	16.2	0.48	3464.7	2.3	18	9
16/17	14	1.45 (20.0)	9.9	19.6	0.51	4000.8	2.8	21	10

AC: autonomous communities; FV: fruits and vegetables F: fruits; V; vegetables.

**Table 2:** Characteristics of the School Fruit and Vegetables Scheme by AC: averages for the 2009–2017 period.

Geographic Area	Ed.	Budget	Stud. (×1000)	Cost × Stud./yr.	Duration	Cost × Stud./Day	Quantity of FV per Stud.	FV Offered × Day × Stud.	Variety		Recommendation of Foods		
									Fruits	Vegetables	Local	Seasonal	Organic
	*n*	millions (%)	*n* (%)	€	days	€	kg	g	*n*	*n*	No. of Editions		
Spain		12,011.3 (100.0)	1334.3 (18.7)	9.0	12.8	0.71	2.5	195	7.0	0.9	8	8	0
Andalucía	8	2154.6 (17.9)	255.2(17.6)	8.4	5.6	1.9	2.2	393	6.8	1.4	5	5	5
Aragón	8	555.8 (4.6)	60.3 (31.5)	9.2	16.3	0.8	2.3	141	6.1	0.0	0	2	0
Asturias	6	290.8 (2.4)	26.0 (21.8)	11.2	33.5	0.6	3.1	93	8.5	0.0	2	3	1
Islas Baleares	8	158.4 (1.3)	27.0 (16.6)	5.9	11.8	1.8	0.9	76	9.5	0.1	5	3	0
Islas Canarias	8	459.4 (3.8)	28.9 (9.5)	15.9	42.4	0.6	5.1	120	8.6	0.4	5	4	4
Cantabria	3	116.7 (1.0)	50.4 (64.0)	2.3	7.0	0.3	0.2	29	8.7	1.0	2	2	2
CLM	8	745.5 (6.2)	49.0 (14.7)	15.2	18.3	1.5	2.1	115	9.5	1.0	5	4	1
CyL	8	700.0 (5.8)	144.0 (45.6)	4.9	9.9	0.8	2.0	202	2.3	0.8	5	5	1
Cataluña	8	2149.2 (17.9)	297.3 (25.2)	7.2	13.0	0.9	3.6	277	13.4	1.8	2	5	0
Extremadura	7	378.2 (3.1)	36.7 (22.4)	10.3	13.9	1.1	2.4	173	7.7	0.3	4	5	0
Galicia	7	256.6 (2.1)	111.3 (32.8)	2.3	17.9	0.2	1.4	78	11.0	0.0	5	5	4
La Rioja	8	216.9 (1.8)	11.2 (24.1)	19.3	70.8	2.2	6.4	90	3.1	1.7	3	2	2
Murcia	8	512.0 (4.3)	43.4 (16.7)	11.8	22.8	1.1	1.6	70	6.5	5.5	5	5	0
Navarra	8	208.2 (1.7)	7.3 (7.4)	28.4	69.9	0.5	8.1	116	1.9	0.3	0	5	0
País Vasco	3	125.0 (1.0)	13.9 (4.4)	9.0	7.0	1.3	0.3	43	4.3	0.0	2	2	2
Valencia	8	1767.9 (14.7)	231.3 (30.9)	7.6	9.7	0.7	1.1	113	3.9	0.5	4	4	0

Ed.: number of editions in which they participated; Stud.: student; yr.: year; FV: fruits and vegetables; CLM: Castilla la Mancha; CyL: Castilla y León.

**Table 3:** Education activities accompanying the School Fruit and Vegetables Scheme for each autonomous community (2009–2017), number of times (first year).

AC	Recreational–Educational Activities	Didactic Materials	Visits	Training Workshops	Cooking Classes	School Gardens
Andalucía	7 (2009)	7 (2009)	1 (2011)	5 (2009)	6 (2010)	1 (2013)
Aragón	4 (2011)	1 (2016)	2 (2015)		1 (2016)	
Asturias		2 (2015)	1 (2016)		1 (2016)	
Islas Baleares	3 (2012)	2 (2010)		1 (2010)	1 (2015)	2 (2015)
Islas Canarias	3 (2012)		2 (2012)	2 (2012)	2 (2012)	
Cantabria						
CLM	3 (2013)	1 (2009)		3 (2009)		
CyL			2 (2015)	1 (2016)	1 (2016)	
Cataluña	3 (2011)	4 (2010)	4 (2010)		1 (2016)	
Extremadura	3 (2014)	2 (2014)	3 (2014)		1 (2016)	2 (2015)
Galicia	1 (2015)	1 (2016)	2 (2013)			
La Rioja	2 (2015)	2 (2015)	2 (2015)		1 (2015)	
Murcia	6 (2010)	3 (2013)	4 (2010)	3 (2010)	1 (2016)	1 (2016)
Navarra	3 (2013)	3 (2014)				1 (2015)
País Vasco	2 (2012)	1 (2013)		2 (2012)		
Valencia	2(2010)	4 (2010)	1 (2016)	5 (2010)		
TOTAL	41	33	24	22	16	7
